# Fast Skeletal Muscle Troponin Activation Increases Force of Mouse Fast Skeletal Muscle and Ameliorates Weakness Due to Nebulin-Deficiency

**DOI:** 10.1371/journal.pone.0055861

**Published:** 2013-02-20

**Authors:** Eun-Jeong Lee, Josine M. De Winter, Danielle Buck, Jeffrey R. Jasper, Fady I. Malik, Siegfried Labeit, Coen A. Ottenheijm, Henk Granzier

**Affiliations:** 1 Department of Physiology, University of Arizona, Tucson, Arizona, United States of America; 2 Institute for Cardiovascular Research, Department of Physiology, VU University Medical Center Amsterdam, Amsterdam, The Netherlands; 3 Research & Early Development, Cytokinetics, Inc., South San Francisco, California, United States of America; 4 Department of Integrative Pathophysiology, Universitätsmedizin Mannheim, University of Heidelberg, Mannheim, Germany; University of Minnesota, United States of America

## Abstract

The effect of the fast skeletal muscle troponin activator, CK-2066260, on calcium-induced force development was studied in skinned fast skeletal muscle fibers from wildtype (WT) and nebulin deficient (NEB KO) mice. Nebulin is a sarcomeric protein that when absent (NEB KO mouse) or present at low levels (nemaline myopathy (NM) patients with NEB mutations) causes muscle weakness. We studied the effect of fast skeletal troponin activation on WT muscle and tested whether it might be a therapeutic mechanism to increase muscle strength in nebulin deficient muscle. We measured tension–pCa relations with and without added CK-2066260. Maximal active tension in NEB KO tibialis cranialis fibers in the absence of CK-2066260 was ∼60% less than in WT fibers, consistent with earlier work. CK-2066260 shifted the tension-calcium relationship leftwards, with the largest relative increase (up to 8-fold) at low to intermediate calcium levels. This was a general effect that was present in both WT and NEB KO fiber bundles. At pCa levels above ∼6.0 (i.e., calcium concentrations <1 µM), CK-2066260 increased tension of NEB KO fibers to beyond that of WT fibers. Crossbridge cycling kinetics were studied by measuring *k_tr_* (rate constant of force redevelopment following a rapid shortening/restretch). CK-2066260 greatly increased *k_tr_* at submaximal activation levels in both WT and NEB KO fiber bundles. We also studied the sarcomere length (SL) dependence of the CK-2066260 effect (SL 2.1 µm and 2.6 µm) and found that in the NEB KO fibers, CK-2066260 had a larger effect on calcium sensitivity at the long SL. We conclude that fast skeletal muscle troponin activation increases force at submaximal activation in both wildtype and NEB KO fiber bundles and, importantly, that this troponin activation is a potential therapeutic mechanism for increasing force in NM and other skeletal muscle diseases with loss of muscle strength.

## Introduction

The skeletal muscle sarcomere is a highly organized and intricate structure that consists of a regular array of actin-based thin filaments that interdigitate with myosin-based thick filaments; these filaments slide past each other as muscles contract, a process driven by the mechano-chemical cycling of the thick-filament based myosin crossbridges [1]. The force level that is generated by the sarcomere depends on the degree to which the thin and thick filaments overlap, the degree of thin filament activation, and on the kinetics of the crossbridge cycle that determine the fraction of the crossbridges that develops force. Recent studies on mouse models deficient in nebulin[2]–[4] indicate that the large filamentous protein nebulin plays an important role in each of these force determinants and, importantly, that when nebulin levels are reduced in nemaline myopathy (NM) patients, severe muscle weakness ensues[5]–[7].

**Figure 1:**
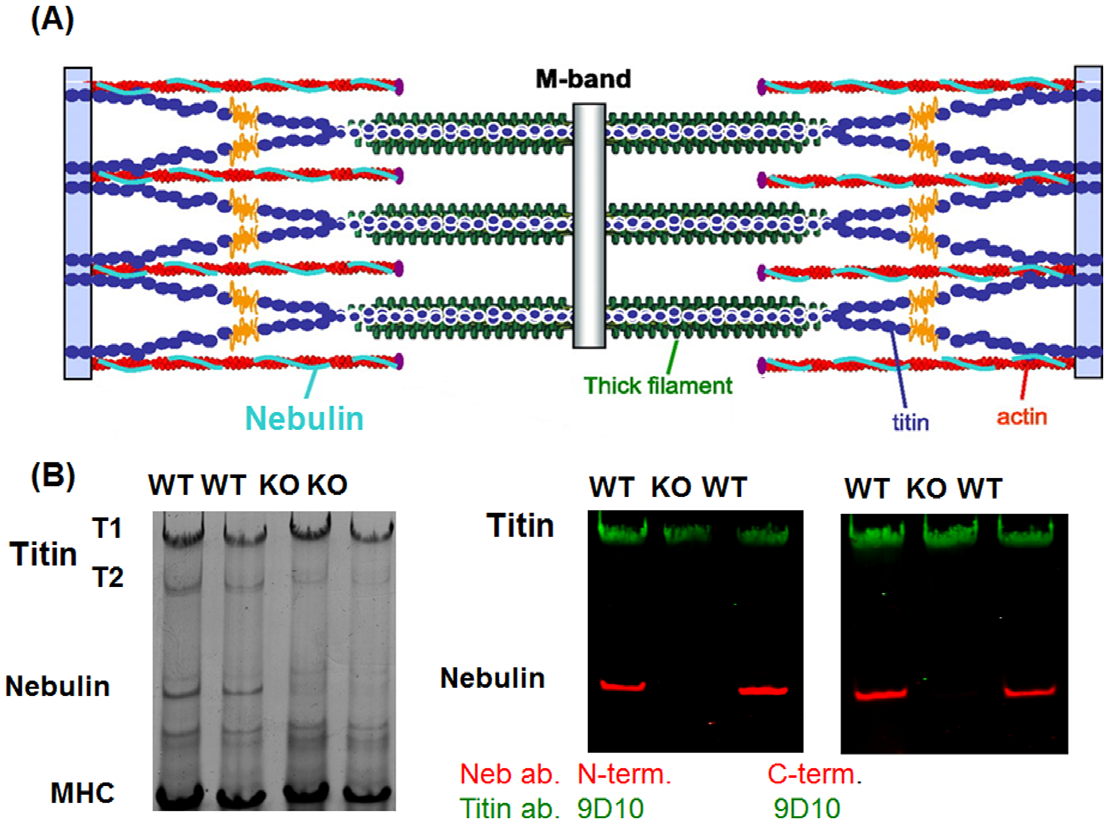
Location of nebulin in the sarcomere and protein expression in WT and NEB KO mouse TC muscle. A) Nebulin is a large sarcomeric protein that is coextensive with the actin filament. B) Protein expression in WT and NEB KO mouse TC muscle. (1% agarose gel). Left coomassie blue stained gel. Right: Westernblot with anti-titin antibody 9D10 (green) and anti-nebulin antibody raised to nebulin’s N-terminus (left) and anti-nebulin antibody raised to nebulin’s C-terminus. Nebulin is a ∼800 kDa protein that is only present in WT mice.

The C-terminus of nebulin is anchored at the Z-disk, the majority of the nebulin filament is co-extensive with the thin filament, and the N-terminus of nebulin is near the pointed end of the thin filament (Fig. 1A) [8]–[11]. The human *NEB* (nebulin) gene is large, containing 183 exons in human that encode a ∼800 kDa protein [12], [13]. The bulk of the molecule is comprised of small modules that are organized into seven-module super-repeats that match the 38.5 nm repeat of the actin filament [12]–[15]. This arrangement allows each nebulin module to interact with a single actin monomer, and each nebulin super-repeat to associate with a single tropomyosin/troponin complex [14], [15]. Recent work indicates that nebulin’s C-terminus interacts with the actin nucleating protein N-WASP, that this interaction plays an important role in skeletal muscle hypertrophy, and that this process is controlled by phosphorylation of nebulin’s serine rich domain [16].

Studies using nebulin KO mouse models have shown that thin filaments are shorter in nebulin deficient muscle, indicating that nebulin contributes to thin filament length specification [3], [4]. Furthermore, on the descending limb of the force-sarcomere length relationship, the shorter thin filaments of nebulin deficient muscle give rise to reduced filament overlap and reduced force [3], [4], [17]. Finally, nebulin plays a role in increasing calcium sensitivity and, importantly, in increasing the fraction of the crossbridges that generates force, by altering crossbridge cycling kinetics [2], [18]. Subsequent studies on biopsies from NM patients support that nebulin’s compromised role in augmenting force generation contributes to the muscle weakness of nebulin-based NM patients [2], [6]. Therapeutic approaches to lessen force impairment in NM patients are currently unavailable.

A class of fast skeletal troponin activators was recently discovered that can augment force development of skeletal muscle by slowing the dissociation rate of calcium from the troponin complex and enhancing crossbridge formation at a given calcium concentration [19]. Given that deficits in force production described above in NM might be improved by this mechanism of action, the purpose of this study was to establish the effect of the fast skeletal troponin activator, CK-2066260, on force generation in 1) skinned fibers from WT mice, and 2) skinned fibers from NEB-KO mice.

## Methods

### Ethics Statement

Experiments were approved by the University of Arizona Institutional Animal Care and Use Committee (protocol 07–090) and followed the U.S. National Institutes of Health “Using Animals in Intramural Research” guidelines for animal use.

### Animal Model

We used wildtype (WT) and nebulin-deficient (NEB KO) mice in which the nebulin gene is inactivated by targeting its promoter region [3]. Inactivation of the nebulin gene and genotyping of mice was as described previously [3]. NEB KO mice die at early age [3] and we were restricted to comparing ∼1 week old WT and NEB KO mice. We focused our studies on the tibialis cranialis (TC) muscle (this muscle is also known as tibialis anterior) which is a fast fiber muscle type that is convenient to use for skinned fiber studies. Mice were anesthetized using isoflurane and the TC muscles were dissected. For the mechanical studies, the muscle was chemically skinned using 1% Triton X-100 in relaxing solution. For protein expression studies, muscles were quick-frozen in liquid nitrogen and stored at −80°C. In addition to PCR genotyping, absence of nebulin expression was also confirmed by 1% agarose protein gels (Fig. 1B shows examples).

### Muscle Preparations

Skinned TC muscles were dissected into muscle fiber bundles (cross-sectional area (CSA) ∼0.02 mm^2^; length ∼1.5 mm) and small aluminum clips were glued at each end of the muscle strip. Note that fiber bundles were used for ease of experimentation (single fibers are only ∼10 µm in diameter). Because the bundles contain different fiber types that express neonatal myosin, MHC type IIA and IIB, and small amounts of MHC type I [2], to know the effect of CK-2066260 on the individual fiber types requires future follow-up studies at the single fiber level. The muscles were attached to a force transducer (model 405A, Aurora Scientific) and a length controller (model 322C, Aurora Scientific) [20]. We used a custom-designed experimental setup (model 802D, Aurora Scientific) with an experimental stage that was mounted on top of an inverted microscope with 8 isolated wells that were pre-loaded with activating solutions that contain a range of calcium concentrations. The fiber bundles were visualized via a CCD camera, and from the video images the sarcomere length was measured on-line using a spatial autocorrelation function (model 901, Aurora Scientific). The experimental stage was temperature controlled at 15°C. The thickness and width of the preparation were measured and the CSA was calculated by assuming an elliptical cross-section. The CSA was used to convert measured forces into tension (in mN/mm^2^).

### Skinned Muscle Solutions

We used relaxing solution (RS), pre-activating solution (Pre-A), and maximal activating solution (AS, pCa 4.5). Sub-maximal activating solutions were obtained by mixing RS and AS according to Fabiato and Fabiato [21]. All solutions contained the following: BES, 40 mM; DTT, 1 mM; creatine phosphate (PCr), 33 mM; creatine phosphokinase (CPK), 200 U/ml; the ionic strength was adjusted to 180 mM with K-proprionate; pH 7.0 at 15°C. Relaxing solution, pre-activating solution and activating solution contains 6.9, 6.7, 6.6 mM MgCL_2_, respectively. For Na-ATP the values were 6.0, 6.0 and 6.2 mM, for EGTA 10.0, 1.0, and 0.0 mM, for Ca-EGTA 0, 0, and 10 mM and for K-proprionate 3.3, 30.4, and 2.1 mM, respectively.

### Fast Skeletal Troponin Activator

CK-2066260 belongs to a class of fast skeletal troponin activators discovered by Cytokinetics [19] and was provided by the Research and Early Development department at Cytokinetics (South San Francisco, CA). CK-2066260 selectively binds to and activates the fast skeletal troponin complex by increasing its sensitivity to calcium. CK-2066260 was added to all experimental solutions (relaxing, pre-activating and activating solutions) from a DMSO stock (final DMSO concentration 1%). Control solutions contained 1% DMSO with no CK-2066260 (vehicle control).

### Tension-pCa Measurements

**Figure 2:**
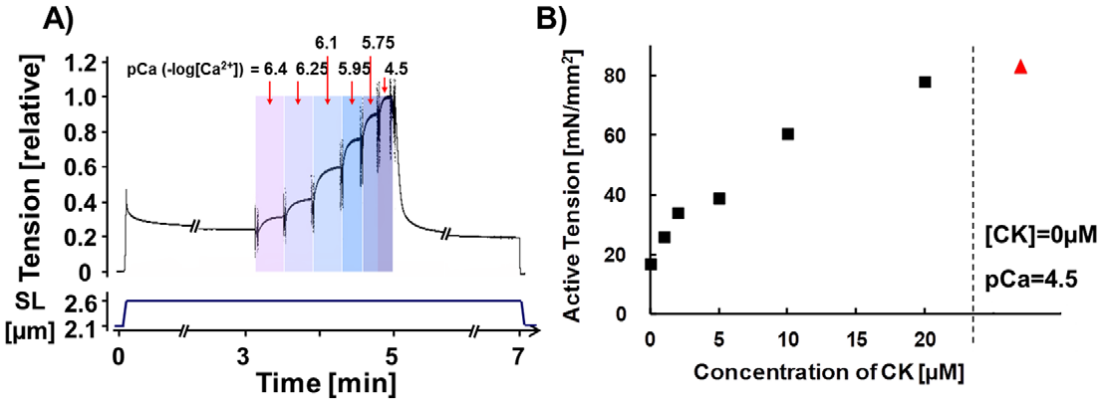
Experimental Protocol and effect of CK-2066260 on active tension at submaximal activation. A) Skinned skeletal muscle fibers (*TC* muscle) were stretched, held for 7 min, and were then released. During the hold phase, the muscle was exposed to various pCa activating solutions, and was then relaxed again. B) Effect of CK-2066260 on active tension at submaximal activation level (pCa = 6.25). Active tensions at a range of CK-2066260 concentrations (black symbols). The maximal active tension (pCa 4.5) at 0 µM CK-2066260 is shown to the right in red.

Fiber bundles while in relaxing solution were stretched to either sarcomere length (SL) 2.1 µm or 2.6 µm, were held at the extended length for 7 min, and were then released. During the hold phase the preparation was first incubated in a pre-activating solution and was then activated in the following sequence pCa 6.4, 6.25, 6.1, 5.95, 5.75 and 4.5 (see Fig. 2A). The muscle was then relaxed again and released. To study the length-dependence of activation (LDA), we first obtained a tension-pCa curve at SL of 2.1 µm, then a relation at 2.6 µm both without CK-2066260 and after that the same sequence was repeated in the presence of 10 µM CK-2066260. Experiments were also performed in which the tension-pCa curves were constructed by activating the fiber bundles at a certain pCa solution (randomly selected) followed by relaxation and a repeat with activation at a different pCa.

Active tension was measured at the plateau of each activation (Fig. 2A). Active tension was normalized by the maximal active tension at pCa 4.5 (T_max_), and plotted against the pCa to determine the tension-pCa curve. The tension-pCa curves were fit to the Hill equation: T/T_max_ (relative tension) = [Ca^2+^]*^n^* ^H/^(K+[Ca^2+^]*^n^* ^H^), where *n*_H_ is the Hill coefficient, and pCa_50_ (pCa value that results in 50% of the maximal activate tension measured at pCa 4.5) was calculated as (-logK)/*n*_H_. In the LDA experiments, we determined the differences between pCa_50_ of the tension-pCa curves measured at SL 2.1 and 2.6 µm and used this as an index of length-dependent activation (i.e. ΔpCa_50_). Rundown was determined from a maximal activation (pCa 4.5; SL 2.1 µm) prior to measurement of the first tension-pCa curve and once more after the last curve (typically we measured 4 curves). Experiments in which rundown was less than 10% were accepted in this study (on average rundown was absent).

### *K*_tr_ Measurement

The rate constant of tension redevelopment (*k_tr_*) was measured at pCa 5.95, 5.75 and 4.5 by using the large release, rapid shortening, rapid re-stretch approach [22]. First, the muscle preparation was activated and once the steady-state was reached, the preparation was rapidly shortened by 20% of the initial length, which reduced tension to zero. This was followed by unloaded shortening lasting 30 msec. The remaining bound crossbridges were mechanically detached by rapidly (1 msec) re-stretching the muscle fiber bundles to its original length, after which tension redevelops. Measurements were performed by controlling fiber length as the quality of the striation patterns did not permit control of sarcomere length. *k_tr_* was determined by fitting the rise of tension redevelopment following re-stretch by a mono-exponential equation: T = T_ss_(1-e^-Ktr.t^), where T is tension at time t, T_ss_ is steady-state tension, and *k_tr_* is the rate constant of tension redevelopment.

### Protein Gels and Westernblots

The anti-titin antibody (9D10 [23], [24]) was obtained from the Developmental Studies Hybridoma Bank, University of Iowa. For details on nebulin antibodies, see acknowledgments. Nebulin antibodies were obtained from Myomedix, Mannheim Germany (www.myomedix.com). For additional technical details, please see[25]–[27].

### Statistical Analysis

Data are shown as mean ± SEM. Paired or unpaired t-tests or ANOVA was used, as appropriate, to test for statistical significance with *p*<0.05.

## Results

We performed tension-pCa studies on skinned muscle fiber bundles from ∼1 week old WT and NEB KO mice (for protocol see Fig. 2A) and studied the effect of the fast skeletal muscle activator CK-2066260 on tension. The initial studies were performed at a sarcomere length (SL) of 2.1 µm because at this sarcomere length WT and NEB KO fibers have a similar degree of filament overlap [2], which simplifies interpretation of results. We first established the CK-2066260 dose-response curve by measuring active tension in skinned WT fiber bundles at pCa 6.25 and adding CK-2066260 at concentrations that ranged from 0–20 µM. Tension in the absence of CK-2066260 was ∼25% of the maximal tension at pCa 4.5 and the addition of CK-2066260 increased tension in a dose-dependent manner (Fig. 2B) with 20 µM CK-2066260 resulting in a tension that was near the maximal tension, T_max_ (pCa 4.5). Thus, in the µM range CK-2066260 is highly effective in increasing submaximal active tensions of wildtype mouse skeletal muscle.

### Effect of CK-2066260 on Calcium Sensitivity of Active Tension

**Figure 3:**
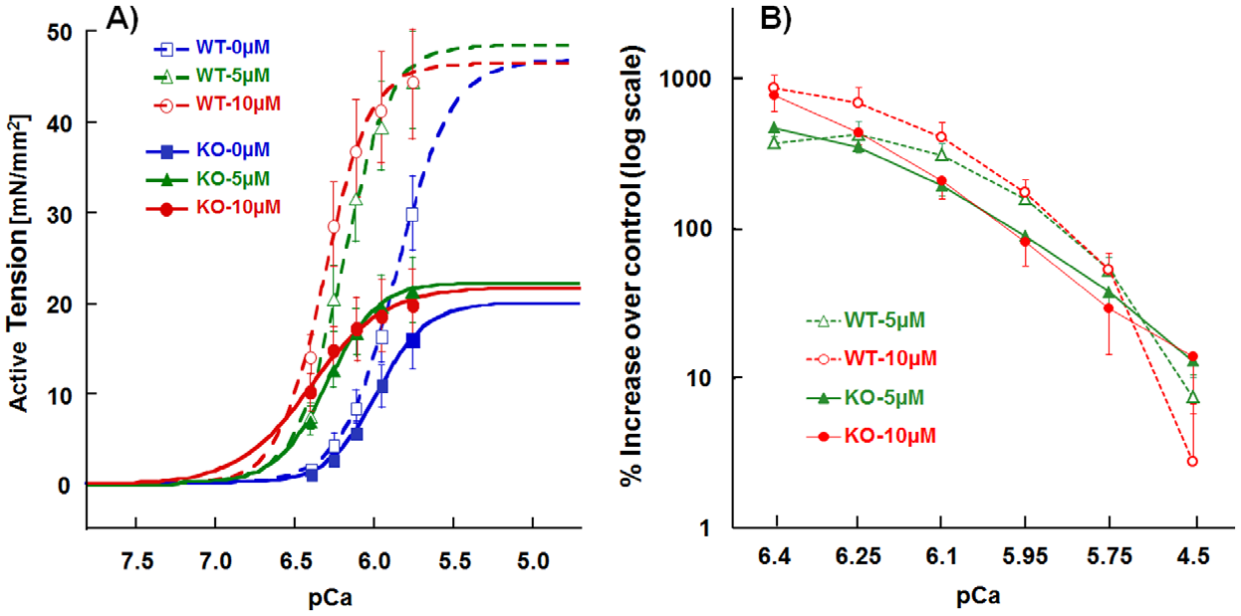
Tension–pCa relations in WT and NEB KO skinned TC muscle fibers. A) Average active tension-pCa curves in WT (open symbols, dashed) and KO (closed symbols, solid) mice with 0 (blue), 5 µM (green) and 10 µM (red) CK-2066260 at SL = 2.1 µm. Curves in CK were shifted to the left in both WT and KO, i.e. calcium sensitivity is increased. Note that at pCa>6.0, CK-2066260 increased active tension of NEB-KO muscle beyond that of WT. B) Relative active tension increase (plotted on a log scale) in 5 and 10 µM of CK over 0 µM of CK at various pCa levels. Regardless of genotype, active tensions were significantly increased from ∼800% at the pCa 6.4 to ∼10% at pCa 4.5. Results from five WT and five KO mice.

We measured the full tension – pCa relations in WT and NEB KO fiber bundles, normalized tensions to T_max_ and used the normalized curves to derive the pCa_50_ (pCa that results in a tension that is 50% of T_max_). T_max_ in KO fiber bundles was only 42% of WT fibers (Table 1, 0 µM CK-2066260). This finding is consistent with previous work on nebulin deficient mouse muscle that reported the tension in KO fibers to be ∼45% of WT fibers [2]. In the present study, calcium sensitivity was found to be increased in the NEB KO (Table 1), whereas previously a reduction was found [2]. Increased calcium sensitivity might be explained by the upregulation of a low level (∼10%) of slow skeletal muscle TnT (sTnT) and slow TnI (sTnI) in nebulin KO TC muscle [2], or a change in their post-translational modification. This notion is supported by experiments in which in both NEB KO and WT fibers the native troponin complex was replaced by recombinant troponin and calcium sensitivity was reduced [2]. Next we studied the effect of CK-2066260 on the tension-pCa curve and selected, based on the measured dose-response curve, 5 and 10 µM CK-2066260. In both genotypes CK-2066260 substantially shifted the tension-calcium relationship to the left as compared to vehicle alone (0 µM CK-2066260), with the most prominent tension increase observed at lower activation levels, while peak tension (T_max_) was not affected (Fig. 3A). To highlight the effect of CK-2066260 on submaximal active tension, we plotted the relative increase in active tension in the presence of 10 µM CK-2066260 over that measured in absence of CK-2066260, at various pCa levels (Fig. 3B). The increase in tension was ∼800% at pCa 6.4 and decreased as calcium concentration increased, with only a small ∼5% increase in WT and a ∼10% increase in KO at pCa 4.5 (p>0.05 compared to the absence of CK-2066260). There were no differences between the genotypes in the relative increase in tension in CK-2066260. In summary, CK-2066260 was highly effective in increasing active tension levels with very large effects at low to intermediate levels of activation.

**Table 1:** T_max_ (tension at pCa 4.5); calcium sensitivity (pCa_50_) and Hill coefficient (n_H_) of WT and KO in 0, 5, and 10 µM CK-2066260.

Genotype/parameter	CK-2066260 concentration		
	0 µM	5 µM	10 µM
WT- T_max_ (mN/mm^2^)	46.6±5.5	49.7±5.1	47.8±6.0
KO- T_max_ (mN/mm^2^)	19.9±4.0**	22.1±4.1**	22.2±4.5**
WT- pCa_50_	5.85±0.04	6.18±0.04# #	6.30±0.04# #√√
Δ from 0 CK (pCa unit)		0.34±0.02	0.45±0.02√√
KO- pCa_50_	5.98±0.03*	6.29±0.01* # #	6.43±0.05# #√√
Δ from 0 CK(pCa unit)		0.31±0.02	0.45±0.04√
WT- n_H_	2.64±0.07	3.21±0.20	3.29±0.34
KO- n_H_	2.83±0.15	3.14±0.38	2.16±0.58
WT- Tmax (SL 2.6 µm)	58.7±6.0	NA	66.7±6.4#
KO- Tmax (SL 2.6 µm)	34.0±5.7**	NA	44.5±8.0**# #
WT- nH (SL 2.6 µm)	2.7±0.28	NA	2.31±0.24
KO- nH (SL 2.6 µm)	3.09±0.11	NA	1.47±0.24*# #

Results are mean ± SEM from 5 WT and 5 KO mice, except for the last 4 rows that are from 6 WT and 6 KO mice.

* comparison WT vs. KO;

# comparison versus 0 µM CK-2066260;

√ comparison versus 5 µM CK-2066260; single symbol p<0.05, double symbol p<0.01. All measurements were at a sarcomere length (SL) of 2.1 µm, except the bottom 4 rows that were at 2.6 µm.

In WT muscle fibers, pCa_50_ was significantly increased by 0.34 and 0.45 pCa units in 5 µM and 10 µM CK-2066260, respectively. In nebulin deficient fibers, a similar increase occurred with values of 0.31 and 0.45 pCa units (Table 1). We also determined the cooperativity of activation (Hill coefficient, n_H_) and found this to be unchanged (Table 1). Thus, CK-2066260 alters the activation characteristics of mouse skinned fibers by greatly increasing calcium sensitivity.

Because all studies were performed in the presence of 1% DMSO (CK-2066260 solvent) we studied whether DMSO itself affects tension. We compared the tension-pCa relation in solutions without and with 1% DMSO (but no CK-2066260) and measured for each muscle preparation multiple tension-pCa relations (as in our CK-2066260 experiments). DMSO did cause a small increase in active tension, but the same relative increase occurred at all pCa levels. Consequently 1% DMSO did not cause a change in pCa_50_ nor in n_H_ (measurement of cooperativity) and is therefore a safe concentration to use in our experiments. Finally, we also evaluated whether the activation protocol with progressively increasing calcium levels (explained in Fig. 2A) gave the same results as when performing experiments in which the tension-pCa curves were constructed by activating the fiber bundles by a single pCa solution (randomly selected) followed by relaxation, and repeating this sequence at different pCa values. Figure S1 shows that both protocols gave the same results.

In summary, CK-2066260 increases tension in both WT and nebulin deficient fibers, with large effects at submaximal activation levels. Thus, CK-2066260 is a highly effective calcium sensitizer.

### Effect of CK-2066260 on Rate Constant of Tension Development (*k_tr_*)

**Figure 4:**
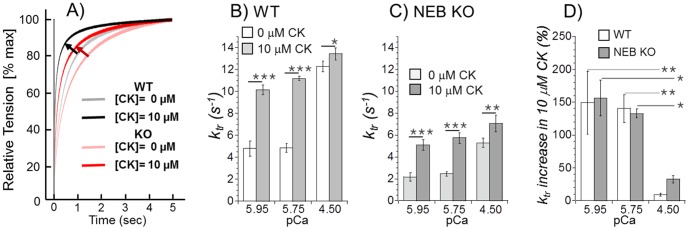
Effect of CK 2066260 on the rate constant of force redevelopment (*k_tr_*). A) Examples of *k_tr_* experiments on WT and NEB KO fiber bundles activated at pCa 5.95 before (control) and after the addition of 10 µM CK-2066260. Shown is the tension recovery phase following the re-stretch (see Methods for details). In the presence of 10 µM CK-2066260 (darker traces) tension recovery is faster than in absence of CK-2066260. B) and C) k*_tr_* results of WT (B) and NEB KO fibers (C). Addition of 10 µM CK-2066260 increases *k_tr_* in both WT and NEB-KO fibers. A two-way ANOVA reveals in WT bundles significant effects of pCa and CK-2066260 on *k_tr_* with a significant interaction between pCa and CK (p<0.001). The same is the case for the KO data, except that the p-value of the interaction term is p = 0.02. D) Change in *k_tr_* in CK 2066260 (as percentage of control values). Results from six WT and six KO mice.

To gain insights in the effect of CK-2066260 on crossbridge cycling kinetics we measured *k_tr_*, the rate constant of force redevelopment following a release/shortening/restretch protocol. This rate constant reflects the sum of the apparent rate constant of conversion of non-force generating to force generating crossbridges, f_app_, and the apparent rate constant of conversion of force generating to non-force generating crossbridges, g_app_ [22]. *k_tr_* was measured at three pCa levels (5.95, 5.75, and 4.5) and the effect of 10 µM CK-2066260 was determined. Fig. 4A shows example experiments and Fig. 4B and 4C summarized results of WT (B) and NEB KO (C) fiber bundles. Under all experimental conditions was *k_tr_* less in NEB KO than WT fiber bundles. *k_tr_* increased with activation level and, importantly, was significantly increased by CK-2066260. The largest effects were present at submaximal activation level where *k_tr_* was increased by more than 150% (Fig. 4D). These results indicate that in WT and NEB KO fibers CK-2066260 alters crossbridge cycling kinetics.

### Length Dependent Effects of CK-2066260

**Figure 5:**
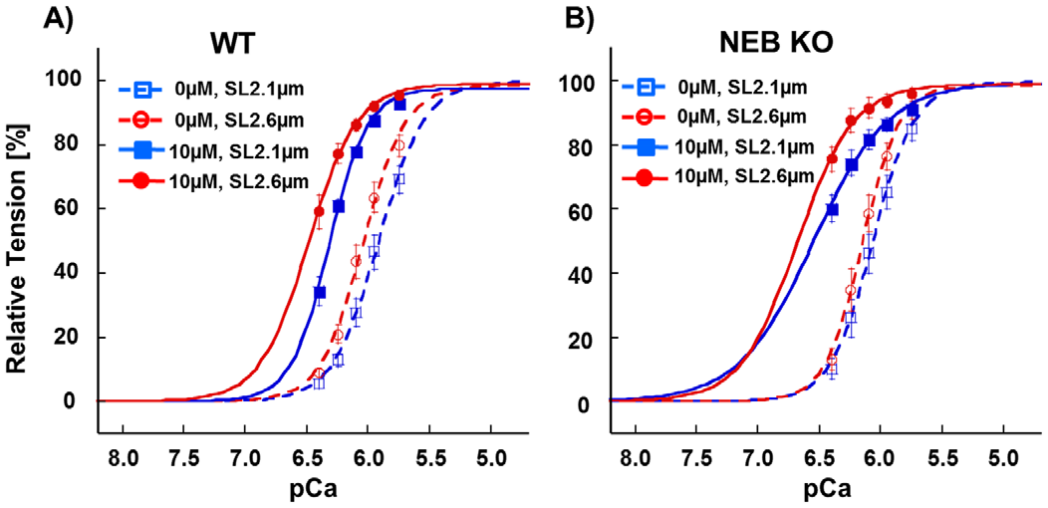
Effect of 10 µM CK-2066260 on tension-pCa curves at short (2.1 µm) and long SL (2.6 µm). Result in WT in A and in NEB KO fibers in B. Increasing SL slightly shifts the curves left-ward (i.e., calcium sensitivity is increased) and CK results in an additional large shift. Results from six WT and six KO mice.

**Figure 6:**
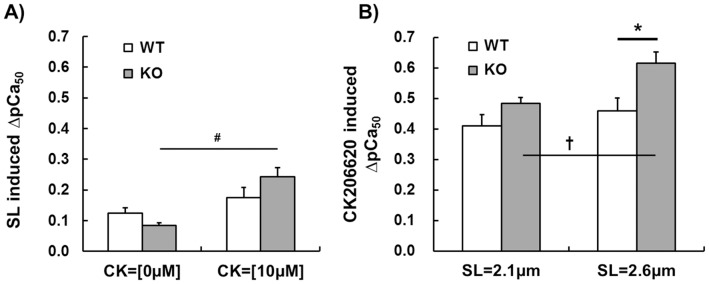
SL-induced ΔpCa_50_ at 0 and 10 µM CK-2066260 and CK 2066260-induced ΔpCa_50_ at SL 2.1 µm and SL 2.6 µm. A) Effect of increasing SL from 2.1 to 2.6 µm on calcium sensitivity (SL-induced ΔpCa_50_) without and with 10 µM CK 2066260. In KO fibers CK-2066260 increased the SL-induced ΔpCa_50_ (#, p<0.05). B) CK-2066260 (10 µM) induced ΔpCa_50_ at SL 2.1 µm and 2.6 µm. Only in KO fibers CK 2066260-induced ΔpCa_50_ was significantly greater at SL 2.6 µm compared to 2.1 µm (†, p<0.05). Furthermore, at SL 2.6 µm CK 2066260-induced ΔpCa_50_ in KO fibers was significantly greater than in WT (*, p<0.05). Results from six WT and six KO mice.

Because muscle operates at a range of sarcomere lengths, a separate set of studies was performed in which tension-pCa curves were measured at SL 2.1 µm and 2.6 µm. To keep rundown to a minimum during the experiment we tested only 10 µM CK-2066260, which required a manageable four force-pCa curves per muscle preparation. An increase in SL caused a leftward shift in the tension-pCa relation (see the small shifts between the pair of broken lines and between the pair of solid lines in Fig. 5), which reflects the well-known phenomenon of length dependence of activation in striated muscle [28]. This SL-induced shift was characterized by determining the ΔpCa_50_ (pCa_50_ at SL 2.6 µm minus pCa_50_ at SL 2.1 µm) and the result is shown in Fig. 6A. The data did not reveal a significant difference between the genotypes. However, within genotype a significant difference was found in the KO fibers as the SL effect was larger in fibers that were exposed to CK-2066260 (Fig. 6A).

We also determined the length-dependence of the CK-2066260 effect on calcium sensitivity by determining the ΔpCa_50_ (pCa_50_ in 10 µM CK-2066260 minus pCa_50_ in 0 µM CK-2066260, see shift between same color broken and solid curves in Figure 5). The results (Fig. 6B) reveal ΔpCa_50_ values of 0.4–0.6 pCa units, which is several times larger than the values obtained by increasing SL (compare Figure 6B with Figure 6A). Thus compared to SL increase, CK-2066260 is a much more efficacious calcium sensitizer. Furthermore, CK-2066260 at a SL of 2.6 µm increases calcium sensitivity to a significantly higher degree in KO fibers than in WT fibers and the CK-2066260 effect on KO fibers is significantly larger at long SL than at short SL (Fig. 6B).

T_max_ was significantly increased by CK-2066260 at the long SL in both genotypes (WT: 58.7±6.0 to 66.7±6.4 mN/mm^2^; KO: 34.0±5.7 to 44.5±8.0 mN/mm^2^ (see Table 1, near bottom). Finally, we also determined the cooperatively of activation (n_H_) at SL 2.6 µm and found that CK-2066260 did not affect n_H_ of WT fibers but that n_H_ was reduced in KO fibers (Table 1, bottom two rows).

## Discussion

Compromised neural input and excitation-contraction coupling is well-known to underlie muscle weakness in many skeletal muscle diseases. However, reduced force production can also result from alterations in sarcomeric proteins [29]. A prime example is nemaline myopathy (NM), the most common non-dystrophic congenital skeletal muscle myopathy [30] that is caused by mutations in six genes that all encode thin filament proteins (TMP2 [31], TMP3 [32], NEB [33], ACTA1 [34], TNNT1 [35], CFL2 [36]). Of these NM genes NEB is most important as it accounts for ∼50 percent of NM cases [33]. Treatment options for NM currently do not exist and gene therapy is still far away. Considering that a hallmark feature of NM is muscle weakness [37] we studied whether the fast skeletal muscle troponin activator CK-2066260 can restore muscle strength in skinned fast skeletal muscle fiber bundles from wildtype and nebulin deficient mice. In both genotypes CK-2066260 causes a large increase in calcium sensitivity of force development with a ∼8-fold increase in force at low activating calcium levels.

CK-2066260 is structurally and functionally closely related to the fast skeletal muscle troponin activator CK-2017357 that is currently in phase II clinical trials for Amyotrophic Lateral Sclerosis [19]. CK-2017357 slows the dissociation rate of calcium from troponin thereby stabilizing the open-conformation of the troponin/tropomyosin complex and enhancing crossbridge formation at a given calcium concentration [19]; the analogue CK-2066260 acts similarly (data not shown). In the present study, we observed that CK-2066260 greatly increases the calcium-sensitivity of force generation with an increase in pCa_50_ of 0.4–0.6 pCa units (Fig. 6B). A large effect is already seen at low doses with a 2 µM dose nearly doubling force at a pCa of 6.25 (Fig. 2B). This effect of CK-2066260 is similar to that of CK-2017357 in human and rabbit fast skeletal muscle fibers [19], indicating that this class of troponin activators is effective in a wide range of species.

Nebulin deficient muscle fibers produced active tensions that were much less than in WT muscle; for example the deficit in T_max_ (maximal active tension at pCa 4.5) was ∼60% at a SL of 2.1 µm (Table 1), which is consistent with earlier findings [2]. Nebulin deficiency results in thin filaments that are on average shorter than in WT muscle [3], [4]; however, it is unlikely that this explains the large tension deficit because the effect of the shorter thin filament length on active tension is minimal at the SL of 2.1 µm where KO fibers reach their optimal sarcomere length and WT fibers are on the ascending limb of the force-sarcomere length relation [2]. An additional known effect of nebulin deficiency that impacts T_max_ is an increase in g_app_ (apparent rate constant with which force generating crossbridges detach) and a decrease in f_app_ (apparent rate constant with which crossbridges enter the force generating state) with the net effect being a reduction in the fraction of crossbridges that generate force [2], [7], [18]. These earlier conclusions were based on an increased tension cost (which reflects g_app_) and a reduction in *k_tr_* in NEB KO fibers (*k_tr_* represents the sum of f_app_ and g_app_ [22]) and a similar *k_tr_* reduction was found in the present study (Fig. 4). We consider it likely therefore that the tension deficit of nebulin KO fibers in the present study has a large contribution from altered crossbridge cycling kinetics.

We found that *k_tr_* increases with activation level (Fig. 4B and C), consistent with the work of others [38]–[40]. This Ca^2+^-dependence of *k_tr_* is likely due to complex kinetic interactions between crossbridge cycling and Ca^2+^-dependent thin-filament dynamics that increase *f_app_* as sub-maximal calcium levels are increased [22], [40]. TnC likely plays an important role in force kinetics since previous studies have provided evidence that TnC mutants with a slower Ca^2+^ dissociation increase *k_tr_* at submaximal activation levels [40], [41]. Similarly, we found that CK-2066260 causes a pronounced increase in *k_tr_* (Fig. 4), consistent with the slowing of Ca^2+^ dissociation from TnC caused by skeletal muscle troponin activators [19]. We propose that the greater calcium sensitivity of the thin filament induced by CK-2066260 increases *k_tr_* because of an increase in *f_app_*. Considering that force is proportional to *f_app_*/(*f_app_*+*g_app_*) (the fraction of cycling crossbridges that develops force [22]), the effect of CK-2066260 will not only be a more rapid rise in force but also an increase in peak force at submaximal activation levels. Thus, both effects are consequences of the increase in calcium sensitivity of the sarcomere caused by CK-2066260.

Our finding that CK-2066260 increased T_max_ at SL 2.6 µm (13% in the WT muscle fibers and 30% in the NEB KO muscle fibers) was unexpected considering that the effect of the activator is thought to be an increase in calcium sensitivity only. The increase in T_max_ suggests that a saturating level of calcium does not cause a maximal number of crossbridges to attach and develop force. It has been reported that even a maximal tetanic contraction in normal healthy muscle has only ∼40% of the available crossbridges attached [34]. If underlying the CK-2066260 induced increase in *k_tr_* at saturating calcium levels (Fig. 4B–D) is solely an increase in *f_app_*, then an increase in T_max_ would be expected. A reduction in the number of force generating crossbridges was previously reported in nebulin-deficient muscle both in the mouse KO model and in NM patients [2]; this finding might underlie the larger effect of CK-2066260 at maximal activation on both *k_tr_* (increase 9% in WT and 34% in KO) and T_max_ (increase 13% in WT and 30% in KO). Finally, the finding that the coefficient of cooperativity (n_H_) is reduced (at 10 µM CK-2066260) in only the NEB KO fibers (Table 1) suggests that the absence of nebulin alters the CK-2066260-thin filament interactions and its downstream effects.

The conclusion that absence of nebulin alters the effect of CK-2066260 on the thin filament is also supported by the length dependency of the CK-2066260 effect on calcium sensitivity. It is well known that increasing SL causes an increase in calcium sensitivity [42], a phenomenon that is especially prominent in cardiac muscle (where it underlies the Frank-Starling mechanism of the heart) but it also occurs in skeletal muscle [43]. In the present study, an increase in SL caused a left-shift in the force-pCa relation of ∼0.1 pCa units, with no difference between the genotypes (Fig. 6A). Although calcium sensitivity is already increased at the long SL, CK-2066260 causes a further increase and this increase exceeds the one at short length (Fig. 6B). At the long SL (2.6 µm) CK-2066260 had a larger effect in NEB KO fibers than in WT fibers (Fig. 6B) which suggests that nebulin deficiency sensitizes troponin to CK-2066260. Although additional studies are required to fully understand the mechanistic basis of these findings, our results establish that CK-2066260 is a potent activator of fast skeletal muscle, especially of nebulin deficient muscle.

In summary, we investigated the effect of the fast skeletal muscle troponin activator CK-2066260 on force development in fast skeletal muscle of wildtype and NEB KO mice. CK-2066260 was found to greatly increase force development at submaximal activation levels. Considering that most muscles during normal activity operate in the submaximal activation regime [44] the beneficial effect of CK-2066260 on *in vivo* muscle force may be substantial. It is also noteworthy that the class of troponin activators to which CK-206620 belongs is specific to fast skeletal troponin and does not affect cardiac muscle [19], which is an important consideration in terms of therapeutic index. The beneficial effect of CK-2066260 on fast skeletal muscle can be well visualized by our finding that at pCa levels above ∼6.0 (i.e., calcium levels <1 µM) CK-2066260 increases the tension of NEB KO fibers to beyond that of WT fibers (compare red and green solid curves in Fig. 3A with blue broken line). We conclude that fast skeletal troponin activators may ameliorate muscle weakness in NM and other patients with compromised muscle strength.

## Supporting Information

Figure S1 **Effect of 10 µM CK-2066260 on tension-pCa curves in WT (dashed) and NEB KO fibers (solid) with a randomized activation protocol (see text for details).** Regardless of genotype, tension-pCa curves were shifted to left with addition of CK-2066260 (blue-0 µM of CK, red-10 µM of CK). Results from 6 WT and 6 KO mice. The change in pCa_50_ between 0 and 10 mM CK-2066260 (ΔpCa_50_) obtained with the ‘randomize’d protocol is 0.46 pCa units, which is the same as that obtained with the protocol in which calcium in progressively increased (as in Fig. 2), see inset.
(TIF)
