# Supplementary figures and images for: Fast Skeletal Muscle Troponin Activation Increases Force of Mouse Fast Skeletal Muscle and Ameliorates Weakness Due to Nebulin-Deficiency
Source: PLoS One. 2013 Feb 20;8(2):e55861. doi: 10.1371/journal.pone.0055861 (PMC3577798; doi:10.1371/journal.pone.0055861)

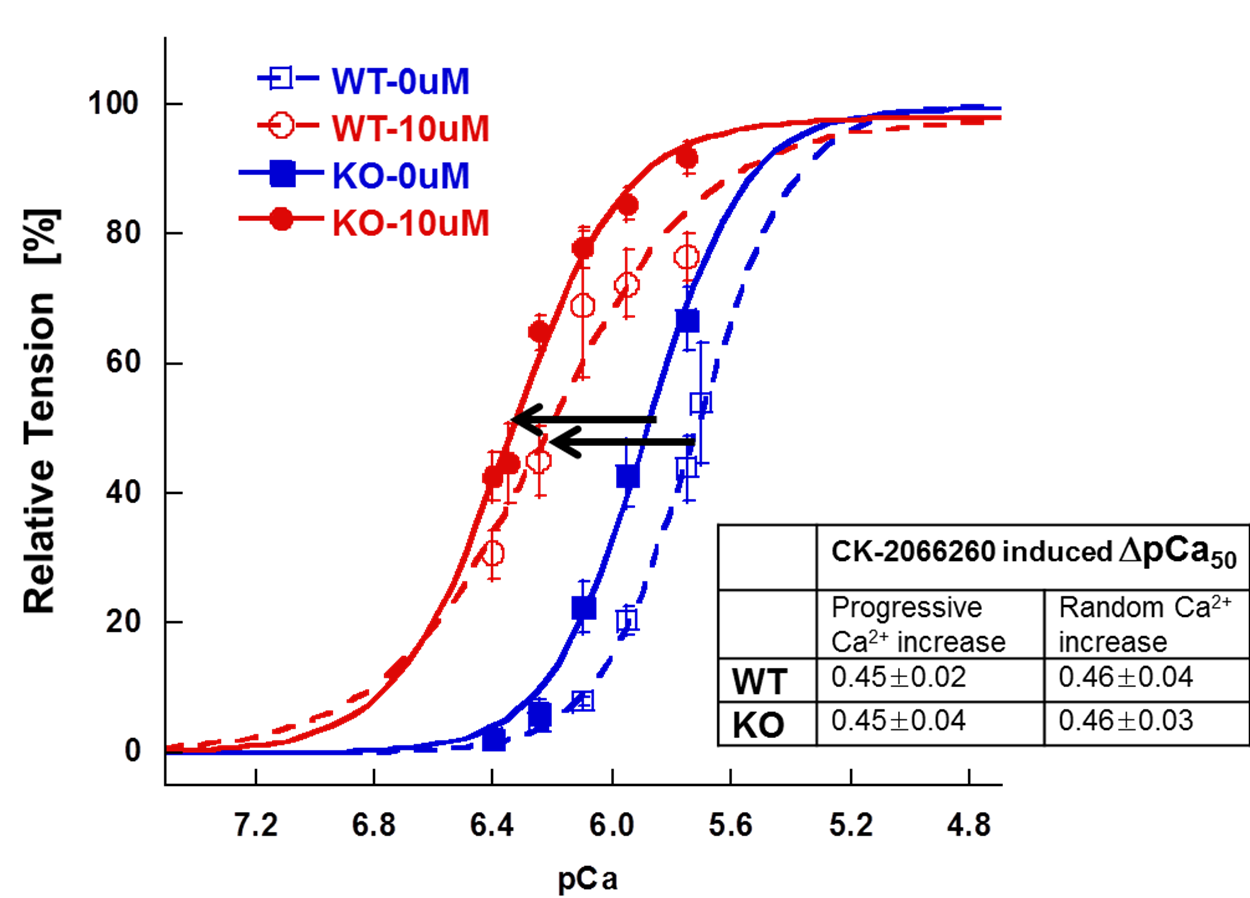

Supplement: Figure S1 — Effect of 10 µM CK-2066260 on tension-pCa curves in WT (dashed) and NEB KO fibers (solid) with a randomized activation protocol (see text for details). Regardless of genotype, tension-pCa curves were shifted to left with addition of CK-2066260 (blue-0 µM of CK, red-10 µM of CK). Results from 6 WT and 6 KO mice. The change in pCa50 between 0 and 10 mM CK-2066260 (ΔpCa50) obtained with the ‘randomize’d protocol is 0.46 pCa units, which is the same as that obtained with the protocol in which calcium in progressively increased (as in Fig. 2), see inset. (TIF) [file pone.0055861.s001.tif]
